# Subclinical inflammation in relation to insulin resistance in prediabetic subjects with nonalcoholic fatty liver disease

**DOI:** 10.1186/s13104-016-2071-x

**Published:** 2016-05-11

**Authors:** Israt Ara Hossain, Salima Akter, Farjana Rahman Bhuiyan, Mijanur Rahman Shah, Mohammad Khalilur Rahman, Liaquat Ali

**Affiliations:** Department of Biochemistry and Cell Biology, Bangladesh University of Health Sciences, Dhaka, Bangladesh; Department of Biotechnology, Bangladesh University of Health Sciences, Dhaka, Bangladesh; Department of Microbiology and Immunology, Bangabandhu Sheikh Mujib Medical University (BSMMU), Dhaka, Bangladesh; Department of Biochemistry and Molecular Biology, University of Dhaka, Dhaka, Bangladesh

## Abstract

**Background:**

Nonalcoholic fatty liver disease (NAFLD) is a metabolic disease commonly associated with obesity, type 2 diabetes, and inflammation-all features of insulin resistant syndrome. However, very limited data are available regarding the association of subclinical inflammation and insulin resistance with NAFLD in a prediabetic state. We, therefore, conducted the study to assess this relationship among this population.

**Methods:**

We studied a cross-sectional analytical design of 140 [male/female, 77/63; age in years (ranges), 45 (25–68)] prediabetic subjects after confirming with 75 g oral glucose tolerance test. The diagnosis of NAFLD was made by ultrasonic examination of the liver and divided into groups of without NAFLD (n = 63) and NAFLD (n = 77). All individuals underwent anthropometric and clinical examinations. Among laboratory investigations, serum glucose was estimated by glucose oxidase method, serum lipid profile and liver enzymes were measured by the enzymatic colorimetric method and glycated hemoglobin was measured by high performance liquid chromatography technique. Serum insulin and high sensitivity C reactive protein (hsCRP) were measured by enzyme immunoassay technique. Insulin resistance (HOMA-IR) was calculated by homeostasis model assessment (HOMA).

**Results:**

There was significantly higher levels of hsCRP (2.82 ± 1.60 vs. 1.39 ± 0.66 mg/l, P < 0.001) and HOMA-IR (4.03 ± 1.39 vs. 1.98 ± 1.04, P < 0.001) in NAFLD subjects compared to their without NAFLD counterparts. hsCRP [odds ratio (OR) = 5.888, 95 % confidence interval (CI) 2.673–12.970, P < 0.001] and HOMA-IR (OR = 4.618, 95 % CI 2.657–8.024, P < 0.001) showed significant determinants of NAFLD after potential confounders of body mass index and triglyceride were adjusted.

**Conclusions:**

Subclinical chronic inflammation and insulin resistance seem to be independent mediators of the association between NAFLD and prediabetes. The data also indicate that the inflammatory condition and insulin resistance are associated with each other and these, in turn, are affected by adiposity and dyslipidemia in prediabetic subjects.

## Background

The entity of ‘prediabetes’, termed by WHO as ‘impaired glucose regulation (IGR)’ is now being recognized as a constellation of three different disorders which may have different isolated or combined pathophysiological characteristics. A study from BIRDEM has shown that Bangladeshi prediabetic population may be categorized into three groups based on their pathophysiological mechanism [1]. These are isolated impaired fasting glucose (I-IFG), isolated impaired glucose tolerance (I-IGT) and a combination of both defects (IFG–IGT). Since insulin resistance (IR) is associated with the majority of the subjects in the IGR and since this state is known to be associated with increased cardiovascular morbidity and mortality [2], it is imperative to investigate this group for the development of nonalcoholic fatty liver disease (NAFLD).

NAFLD is becoming a major public health problem all over the world with increasing incidence of diabetes mellitus, hypertension, dyslipidemia, hepatic steatosis and metabolic syndrome (MS). The prevalence of the disease is expected to increase worldwide and the trend in developing countries is switching towards western lifestyles. The exact etiology of NAFLD is not yet known, but IR is the key mechanism most commonly associated with the pathogenesis of this disorder. This, in turn, seems to create a linkage between NAFLD and type 2 diabetes (T2DM) by subclinical inflammation as a key factor [3]. Although the role of IR in the relationship between subclinical inflammation and NAFLD among the T2DM subjects is fairly well established [2], however, examining this association in a prediabetic state is limited. One study has been done in an urban population of South India and they showed a higher prevalence of NAFLD (54.5 %) among the T2DM subjects compared to those with prediabetes (IFG or IGT) (33 %), I-IGT (32.4 %), I-IFG (27.3 %) and normal glucose tolerance (NGT) (22.5 %) [4]. However, they did not see the association of IR and subclinical inflammation as a reproducible factor in the development of NAFLD.

The pathogenesis of NAFLD leading to metabolic abnormalities has not been fully elucidated. It is characterized by excessive accumulation of free fatty acid within the liver where they are reesterified with glycerol to produce triglyceride (TG). This lipid abnormality leads to the condition of IR by alterations in insulin receptors of peripheral tissues. Increased deposition of fatty acids within the hepatocytes causes oxidative stress with the release of free radicals leading to ATP depletion, mitochondrial dysfunction and hepatic injury-all contribute to the development of inflammation followed by fibrosis [5]. The hepatic steatosis caused by oxidative stress and their underlying mechanism for the development of inflammation still remains unclear. Recent studies revealed that defective insulin action reduces the lipolysis of hepatic fat by decreasing β-oxidation which enhances the release of nonesterified fatty acid (NEFA) from adipose tissue so that more NEFA are transported through the bloodstream and taken up by the liver. In a secondary event of hepatic fat accumulation causes oxidative stress and mitochondrial dysfunction thereby releasing of reactive oxygen species (ROS) through lipid peroxidation and the generation of proinflammatory cytokines leading to inflammation [6].

High sensitivity C reactive protein (hsCRP), a proinflammatory cytokine released during the condition of nonalcoholic steatohepatitis (NASH), also a sensitive marker of systemic inflammation, has been shown to be increased in a hyperglycemic sate. Serum hsCRP levels are elevated in subjects with prediabetes or T2DM. Prior clinical studies showed increased levels of hsCRP as a significant risk factor for the progression of future diabetes [7]. Relationship of inflammatory cytokines with elevated blood glucose levels and the role of IR for this association are available. However, it remains unclear whether a relationship exists between hsCRP and IR with NAFLD in a prediabetic state. Nevertheless, data relating the association of subclinical inflammation with NAFLD in prediabetes is limited, therefore, we hypothesized that a high proportion of prediabetic subjects develop NAFLD and it, in turn, is mediated by subclinical chronic inflammation and IR.

## Methods

### Study design and subjects

A cross-sectional analytical study with group comparison design was conducted during November 2012 to March 2013 in a diabetes care Hospital in Bangladesh (BIRDEM) and a total number of 140 (one hundred and forty) prediabetic subjects were recruited in the study. Diabetes and prediabetes were diagnosed following WHO Group Study criteria [8]. Of the total, upper abdomen ultrasonogram had done and the subjects were divided into 77 without NAFLD and 63 NAFLD groups. We excluded subjects with known liver diseases like viral hepatitis, hepatobiliary diseases, malignancies, inflammatory bowel disease, current medication with lipid and glucose lowering drugs, chronic cardiac, renal and respiratory diseases, stroke, type 1 diabetes, recent change (≥10 %) in body weight and pregnant women. All subjects underwent anthropometric measurements of body weight, height, waist and hip circumference (WC and HC), systolic and diastolic blood pressures (SBP and DBP) were measured by standard procedures. Body mass index (BMI) of the subjects was calculated using the formula of BMI = Weight (kg)/Height (m^2^).

### Biochemical analysis

After overnight fasting (8–14 h) study subjects underwent fasting blood sampling by venipuncture to assess the biochemical tests including fasting and postprandial serum glucose, glycosylated hemoglobin (HbA_1c_), total cholesterol (TC), triglyceride (TG), and high density lipoprotein cholesterol (HDL-c), glutamate oxaloacetate transaminase (GOT), glutamate pyruvate transaminase (GPT), gamma glutamate transaminase (GGT) and alkaline phosphatase (ALP). All tests were measured by standard laboratory methods using a conventional automated analyzer (Dimension^®^ clinical chemistry system, Siemens Healthcare Diagnostics Inc. USA). Low density lipoprotein cholesterol (LDL-c) was calculated by Friedwald formula [9]. Serum insulin and hsCRP were determined by ELISA technique using commercial kits (DRG-International, Germany) and their optical density (OD) were measured by ELISA plate reader (Multiscan FC, USA). The inter- and intra-assay coefficient of variation (% CV) for FSG, insulin and hsCRP were 3.35, 4.33 and 5.12 % and 2.1, 3.11 and 4.01 % respectively. Wintergreen method was used to determine the blood erythrocyte sedimentation rate (ESR) [10]. Homeostatic model assessment insulin sensitivity (HOMA %S) and pancreatic β-cell function (HOMA %B) was derived from fasting values of serum glucose and insulin. Homeostatic model assessment insulin resistance (HOMA-IR) was calculated according to the HOMA model formula: HOMA-IR = fasting insulin × fasting glucose, divided by 22.5 [11]. Total body fat was determined by bioimpedometry.

### Radiological evaluations

Ultrasound imaging of the liver was carried out by a trained radiologist who was blinded of the aims of the study using a high resolution sonography machine (Philips Ultrasound-Ay-MNT-15 TTK, HDI-4000, Netherland) having a 3.5 MHz linear transducer frequency in fasting state of the subjects to assess the degree of steatosis. The presence of fatty liver was confirmed in the absence of alcohol intake by comparative assessment of echoes brightness arising from the hepatic parenchyma with a high level of liver-kidney differentiation, the entrance of echoes into the deepest part of the liver and clear images of the liver blood vessels. NAFLD evaluation was based on scoring the scale as Grade 0: absent (normal echogenicity), Grade 1: mild steatosis, Grade 2: moderate steatosis, Grade 3: severe steatosis [12].

### Statistical analysis

Data of all parameters were presented as the mean ± standard deviation (SD) or number as appropriate. Comparison of mean values between two groups was tested using student’s unpaired *T* test. According to the distribution of data, we used the natural logarithmic transformation of the ESR skewed values for statistical analysis [13, 14]. The sample size was calculated by using the regression model for individual predictors and it depends on the desired power (l−α), significance level (α), the number of predictors and the expected effect sizes. Sampling weights were used by using the formula of N > 50 + 8 m, where m is the number of independent variables (IVs) for testing the multiple correlation and N > 104 + m for testing individual predictors [15]. In our study, there were four IVs (Table 3) and the calculated sample number was 50 + 8 (4) = 82 cases and 104 + 4 = 108 cases for testing individual predictors. These calculations were based on significance level of 5 % (α = 0.05) and 80 % power (P = 0.20). Pearson’s regression curve was done to see the correlation of hsCRP with IR in the study subjects. A multiple linear regression analysis was done to investigate the relationship of HOMA-IR with hsCRP in NAFLD subjects after adjusting the effects of major confounding variables of BMI, WHR, TG, and hsCRP respectively. Multivariate logistic regression was used to predict significant determinants of NAFLD (without NAFLD considered as reference) as the dependent variable and BMI, TG, HOMA-IR and hsCRP as independent variables. To evaluate the effects of HOMA-IR and hsCRP with NAFLD, adjusted odds ratio (ORs) and 95 % confidence interval (CI) estimated by controlling the other significant predictors of NAFLD [16]. A P < 0.05 was considered as statistically significant. All statistical measures were performed using statistical package for social science (SPSS) for windows version 17.0 (SPSS Inc., Chicago, IL, USA).

## Results

The general characteristics of the prediabetic subjects are shown in Table 1. Of the 140 subjects, 54 had IFG (38.6 %, M/F 35/19), 36 had IGT (25.7 %, 12/24) and 50 had IFG-IGT (35.7 %, 30/20) respectively. After liver ultrasound, 63 had NAFLD (45 %, 35/28) and 77 had without NAFLD (55 %, 42/35) respectively. Among fatty liver severity, 73 (52.1 %) had grade 0, 50 (35.7 %) had grade 1, 14 (10.0 %) had grade 2 and 3 (2.1 %) had grade 3 steatosis. Subjects with prediabetes had higher levels of hsCRP, ESR and HOMA-IR. Sociodemographic, anthropometric, clinical and biochemical characteristics of the study subjects according to their fatty liver group are shown in Table 2. Prediabetic subjects with NAFLD had significantly higher levels of WC (P = 0.034), WHR (P = 0.004), SBP (P = 0.011), PPSG (P = 0.042), TC (P = 0.010), TG (P = 0.047), LDL-c (P = 0.014), SGOT (P = 0.026), GGT (P = 0.003), hsCRP (P < 0.001), log ESR (P = 0.001), FSI (P < 0.001), PPSI (P < 0.001) and HOMA-IR (P < 0.001) compared to without NAFLD group. On the other hand, prediabetic subjects with NAFLD, showed significantly lower levels of HDL-c (P = 0.024), HOMA %S (P ≤ 0.001) and HOMA %B (P = 0.007) as compared to their without NAFLD counterparts.

**Table 1:** General characteristic of the study subjects

Parameter	Prediabetic subjects (n = 140)
Sex [n (%)]	
Male	77 (55.0)
Female	63 (45.0)
Age (years)	45.4 ± 9.4
BMI (kg/m^2^)	26.06 ± 4.51
WC (cm)	90.4 ± 8.2
HC (cm)	96.3 ± 8.7
WHR	0.93 ± 0.04
% BF	29.7 ± 7.4
SBP (mmHg)	120 ± 23
DBP (mmHg)	82 ± 20
Different subgroups of prediabetes [n (%)]	
IFG	54 (38.6)
IGT	36 (25.7)
IFG-IGT	50 (35.7)
NAFLD evaluation [n (%)]	
Without NAFLD	77 (55.0)
With NAFLD	63 (45.0)
Grade 0	73 (52.1)
Grade 1	50 (35.7)
Grade 2	14 (10.0)
Grade 3	3 (2.1)
FSG (mmol/l)	5.86 ± 0.45
PPSG (mmol/l)	8.15 ± 1.51
HbA_1c_ (%)	5.85 ± 0.56
TC (mg/dl)	193 ± 39
TG (mg/dl)	163 ± 80
HDL-c (mg/dl)	37 ± 7
LDL-c (mg/dl)	122 ± 35
GOT (IU/l)	31 ± 16
GPT (IU/l)	35 ± 16
GGT (IU/l)	32 ± 14
ALP (IU/l)	102 ± 27
HsCRP (mg/l)	2.04 ± 1.37
log ESR (mm/h)	1.31 ± 0.22
FSI (µIU/ml)	11.26 ± 7.14
PPSI (µIU/ml)	68 ± 41
HOMA %S	55 ± 21
HOMA %B	120 ± 35
HOMA-IR	2.91 ± 1.58

Results are expressed as number (percentage), mean ± SD; level of significance was calculated by Student’s‘t’ test; *n* number of subjects

**Table 2:** Sociodemographic, clinical, anthropometric and biochemical indexes of prediabetic subjects without and with NAFLD

Parameter	Without NAFLD (n = 77)	With NAFLD (n = 63)	*t/*P value
Different subgroups of prediabetes [n (%)]			
IFG	33 (42.9)	21 (33.3)	–
IGT	20 (26.0)	16 (25.4)	–
IFG-IGT	24 (31.2)	26 (41.3)	–
Gender [n (%)]			
Male	35 (55.6)	42 (54.5)	–
Female	28 (44.4)	35 (45.5)	–
Age (years)	44.2 ± 9.4	46.1 ± 9.2	−1.666/0.098
BMI (kg/m^2^)	25.54 ± 4.75	26.68 ± 4.16	−1.489/0.139
WC (cm)	89 ± 8	92 ± 8	−2.137/0.034
HC (cm)	95 ± 9	97 ± 8	−1.038/0.301
WHR	0.93 ± 0.04	0.94 ± 0.04	−2.098/0.038
% BF	29.1 ± 7.2	30.5 ± 7.6	−1.094/0.276
SBP (mmHg)	115 ± 18	126 ± 25	−2.899/0.004
DBP (mmHg)	78 ± 18	87 ± 20	−2.562/0.011
FSG (mmol/l)	5.88 ± 0.49	5.84 ± 0.41	0.534/0.594
PPSG (mmol/l)	7.92 ± 1.51	8.44 ± 1.47	−2.049/0.042
HbA_1c_ (%)	5.81 ± 0.62	5.89 ± 0.47	−0.879/0.381
TC (mg/dl)	185 ± 37	202 ± 40	−2.603/0.010
TG (mg/dl)	151 ± 72	177 ± 88	−1.925/0.047
HDL-c (mg/dl)	38 ± 7	35 ± 7	2.282/0.024
LDL-c (mg/dl)	116 ± 32	130 ± 36	−2.488/0.014
GOT (IU/l)	28 ± 12	34 ± 18	−2.255/0.026
GPT (IU/l)	33 ± 16	37 ± 17	−1.780/0.077
GGT (IU/l)	28 ± 14	36 ± 13	−3.059/0.003
ALP (IU/l)	103 ± 27	100 ± 26	0.516/0.606
HsCRP (mg/l)	1.39 ± 0.66	2.82 ± 1.60	−7.098/<0.001
log ESR (mm/h)	1.25 ± 0.20	1.38 ± 0.22	−3.393/0.001
FSI (µIU/ml)	6.83 ± 2.68	16.68 ± 7.17	−11.132/<0.001
PPSI (µIU/ml)	55 ± 38	83 ± 40	−4.294/<0.001
HOMA %S	59 ± 24	48 ± 15	3.310/<0.001
HOMA %B	127 ± 36	111 ± 32	2.721/0.007
HOMA-IR	1.98 ± 1.04	4.03 ± 1.39	−9.923/<0.001

Results are expressed as number (percentage), mean ± SD; *n* = number of subjects; the level of significance at P < 0.05

**Fig. 1:**
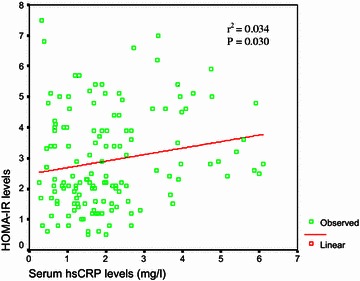
Relationship of hsCRP with HOMA-IR among the total study subjects: serum hsCRP showed significant positive correlation (P = 0.030) with HOMA-IR in the study subjects

Regression curve analysis showed significant positive correlation of HOMA-IR with hsCRP (r^2^ = 0.034, P = 0.030) in the total study subjects (Fig. 1).

Association of HOMA-IR with hsCRP in NAFLD subjects are shown in Table 3. In multiple linear regression analysis, HOMA-IR showed significant positive association with BMI (β = 0.294, P = 0.016), WHR (β = 0.259, P = 0.036) and hsCRP (β = 0.262, P = 0.034) after adjusting the effect of major confounder of TG.

**Table 3:** Multiple linear regression analysis using HOMA-IR as dependent variable after adjusting the effects of major confounders

Parameter	Regression coefficient (β)	t value	P value	95 % Confidence Interval	
				Lower	Upper
BMI (kg/m^2^)	0.294	2.483	0.016	0.019	0.178
WHR	0.259	2.144	0.036	0.564	16.449
TG (mg/dl)	0.015	0.127	0.899	−0.004	0.004
hsCRP (mg/l)	0.262	−2.167	0.034	−0.440	−0.017
CONSTANT	–	−1.597	0.116	−13.643	1.536

Dependent variable: HOMA-IR, Adjusted R^2^ = 0.225; the level of significance at P < 0.05

Association of hsCRP and HOMA-IR with NAFLD group considering without NAFLD as reference after adjusting the effects of major confounding variables are shown in Table 4. In binary logistic regression analysis, hsCRP (OR = 5.888, CI 2.673–12.970, P < 0.001) and HOMA-IR (OR = 4.618, 95 % CI 2.657–8.024, P < 0.001) were found to be significant determinants of NAFLD after adjusting the effects of major confounding variables of BMI and TG respectively.

**Table 4:** Factors associated with NAFLD (considering without NAFLD as reference) after adjusting the effects of major confounders by binary logistic regression model

Parameter	Regression coefficient (β)	SE	P value	Odds ratio	95 % CI of OR	
					Lower	Upper
BMI (kg/m^2^)	0.108	0.069	0.117	1.114	0.973	1.276
TG (mg/dl)	0.003	0.003	0.303	1.003	0.997	1.010
hsCRP (mg/l)	1.773	0.403	<0.001	5.888	2.673	12.970
HOMA-IR	1.530	0.282	<0.001	4.618	2.657	8.024
CONSTANT	−11.331	2.577	0.000	0.000	–	–

*SE* standard error, *CI* confidence interval

Dependent variable: Group (NAFLD vs. without NAFLD as reference); Adjusted R^2^ = 0.565; the level of significance at P < 0.05

## Discussion

The study demonstrates the first cross-sectional study where the relationship between subclinical inflammation and IR with NAFLD has been evaluated among Bangladeshi prediabetic subjects. In this study, 44 % of the prediabetic subjects were affected by NAFLD after liver ultrasound scanning which is in accordance with the prior clinical studies [17, 18] where prevalence also higher, however, their study subjects were previously detected NAFLD from where the prevalence was calculated. Although conventional view holds that NAFLD is directed related to IR and pre-diabetes, emerging evidence indicate that a direct cause-effect relationship may not exist between these pathological conditions [19]. Nevertheless, very limited data are available regarding the prevalence of NAFLD among the prediabetic subjects. Agarwal et al. [20] showed the prevalence of NAFLD after liver ultrasonography among T2DM subjects was 57.2 %. Ruckert et al. [21] found these higher numbers among nondiabetic participants though their fatty liver assessment was based on the abnormal concentration of liver enzymes. They found the fatty liver prevalence in NGT was 18.7 %, IFG 34.6 %, IGT 33.0 %, IGT/IFG 33.3 %, NDD 42.5 % and known diabetes 25.8 % respectively.

hsCRP is an acute phase reactant protein with a short life of around 18 h, and its level is increased during NASH causing low-grade systemic inflammation of the liver. Because of its pro-inflammatory characteristics, hsCRP has been considered as a potential biomarker of subclinical inflammation [7]. Recent data from case and control studies showed elevated serum levels of hsCRP in NAFLD subjects and their association also increased with increasing the severity of fatty liver [20, 21]. However, reports from few studies failed to show the relationship of increased levels hsCRP with the different grades of NAFLD. The current study showed higher hsCRP and IR levels in the study subjects and their strong association with NAFLD compared to the without NAFLD group. Our study results are in line with previous cross-sectional studies that revealed a strong association between the hsCRP and NAFLD in Japanese and Korean Asians [22, 23]. A case–control study by Koruk et al. [24] reported that measurement of the degrees of hsCRP could be used as a simple and sensible technique for the diagnosis of NASH and their relationship with histopathological findings of the liver. In addition, Yoneda et al. [25] from their observational studies reported increased levels of hsCRP can be considered the key mechanism of NAFLD development and its rapid progression to NASH due to oxidative stress. Conversely, data from Wieckowska et al. [26] diverge from our previous findings and they found no significant role of hsCRP in the prognosis of NAFLD.

NAFLD is a hepatic manifestation where IR is the key factor due to abnormal lipid metabolism which in turn progress to hepatic injury caused by oxidative stress. The molecular mechanism of IR in the development of NAFLD and the role inflammatory cytokines for their association is controversial. Recent epidemiological evidence reveals IR as a detrimental factor in the pathogenesis of NAFLD and progression to future NASH, particularly in individuals of obese diabetics and related disorders [24]. It has been suggested that genetic factors that reduce insulin sensitivity and increase the accumulation of triacylglycerol levels which ultimately responsible for the development of IR. Our study supports the association between IR and NAFLD as represented by the fact that all the subjects studied are glucose intolerance. Several studies showed the relationship between IR and NAFLD in subjects with diabetes [24–26] however, this is the first study showing the independent roles of IR and subclinical inflammation in the development of NAFLD among Bangladeshi prediabetic subjects.

The abnormal lipid metabolism in the liver due to IR and their consequence in the pathogenesis of NAFLD is not well elucidated, excessive accumulation of triglycerides within the hepatocytes induces the release of ROS resulting oxidative stress, depletion of mitochondrial ATP production, release of pro-inflammatory cytokines-all of these triggers the subclinical chronic inflammation. When hepatocellular damage occurs, macrophage derived inflammatory cytokines (TNF-α and IL-6) are released leading to the production of acute phase reactant protein (hsCRP) [26]. Increased levels of serum hsCRP reveal the manifestation of IR symptoms like obesity, T2DM and cardiovascular morbidity and mortality. Adipose tissue activates the macrophage infiltration thereby releasing IL-6 and is closely linked to the production of hsCRP, while TNF-α only show its strong relation with IR occurring both in intra-abdominal and subcutaneous tissues [27, 28]. The pathology of NAFLD in the context of obesity is complicated. It appears that dysregulated adipose remodeling and its subsequent chronic inflammation can be considered as the key etiological factors in the development of fatty liver demonstrated from the experimental observations in ob/ob mice after induction of a high-fat diet [29, 30]. However, NAFLD itself is a cause or consequence of IR is not well determined. The lipotoxicity effect of liver impairs the insulin action resulting hyperinsulinemia and subsequent leads to the condition of oxidative stress following chronic inflammation and fibrosis. The association between hsCRP and IR with NAFLD also remained consistent by binary logistic regression analysis when other covariates are adjusted, which reinforces the concept that IR is the consequence of hepatic fat accumulation that in turn leads to the development of subclinical inflammation among prediabetic subjects.

The study has several limitations and recommendations. NAFLD examination was done by ultrasonography imaging of the liver but not by liver biopsy which considered as the gold standard method. Correlation between the different stages of NAFLD (by the histologic picture) and the levels of serum inflammatory markers could not be done. Among inflammatory cytokines, the diagnosis of serum hsCRP provides the most promising results revealed by several cross-sectional studies, however, epidemiological study from larger cohort of prediabetes having NAFLD should be considered to reformulate the relationship between serum inflammatory markers with insulin resistance and their causal association with NAFLD. It is an analytical study with a cross-sectional design thus; no causal association between hsCRP and other interacting molecules with NAFLD could be explored.

## Conclusions

From the above study, it may conclude that a high proportion (more than one-third) of the prediabetic subjects has NAFLD and the distribution of the disorder is almost similar in various subgroups of prediabetes. Insulin resistance followed by subclinical chronic inflammation can be considered as key factors in the pathogenesis of NAFLD among prediabetic subjects. The data also indicate that the inflammatory condition and insulin resistance are associated with each other and those, in turn, are affected by adiposity and dyslipidemia in prediabetic subjects.

## Abbreviations

T2DM: type 2 diabetes mellitus
NAFLD: nonalcoholic fatty liver disease
NASH: nonalcoholic steatohepatitis
HbA_1c_: glycosylated hemoglobin
HPLC: high performance liquid chromatography
hsCRP: high sensitivity C reactive protein
ELISA: enzyme-linked immunosorbent assay
IGR: impaired glucose regulation
TNF-α: tumor necrosis factor alpha
ROS: reactive oxygen species
H_2_O_2_: hydrogen peroxide
DNA: deoxyribonucleic acid
BMI: body mass index
WC: waist circumference
HC: hip circumference
WHR: waist to hip ratio
%BF: percent body fat
SBP: systolic blood pressure
DBP: diastolic blood pressure
IFG: impaired fasting glucose
IGT: impaired glucose tolerance
MS: metabolic syndrome
FSG: fasting serum glucose
PPSG: postprandial serum glucose
TC: total cholesterol
HDL-c: high density lipoprotein-cholesterol
LDL-c: low density lipoprotein-cholesterol
GOT: glutamate oxaloacetate transaminase
GPT: glutamate pyruvate transaminase
GGT: gamma glutamate transaminase
ALP: alkaline phosphatase
log ESR: logarithmically transformed erythrocyte sedimentation rate
FSI: fasting serum insulin
PPSI: postprandial serum insulin
HOMA %S: insulin sensitivity assessed by homeostasis model assessment
HOMA %B: β cell function assessed by homeostasis model assessment
HOMA-IR: homeostasis model assessment insulin resistance
